# Supplementary material for: A mixed methods evaluation of a World Health Organization competency-based training package for foundational helping skills among pre-service and in-service health workers in Nepal, Peru and Uganda
Source: Glob Ment Health (Camb). 2023 Aug 14;10:e55. doi: 10.1017/gmh.2023.43 (PMC10579647; doi:10.1017/gmh.2023.43)

**SUPPLEMENT**

**Supplement 1, Box S1.** Iterative development of manual content for training foundational helping competencies using a competency-based approach for pilot testing in Nepal, Perú and Uganda.

| **Phase 1: Iterative module development (Aug-Nov. 2020)**  Modules went through stages of development prior to pilot testing to iteratively incorporate through rounds of feedback and development. The overall process occurred over four months, as follows:  **Month 1**.   - A group (n=13) of field and academic experts were identified as peer-reviewers for the iterative manual development process. - Informed by the first three items of the ENACT tool, an outline for the initial three FHS modules was drafted. After review by the EQUIP team at George Washington University and World Health Organization, the three modules were fully developed and formatted.   **Month 2**.   - The three modules were sent to the peer reviewers for feedback on content, structure, and formatting. Feedback was collated. - Feedback was incorporated to finalise the initial three modules. Feedback was incorporated to inform the outline and module development for the fourth and fifth modules.   **Month 3**.   - The fourth and fifth FHS modules, based on ENACT items five and six, were sent out to peer reviewers for feedback. Feedback was collated. - Feedback was incorporated to finalise the fourth and fifth modules. Feedback was incorporated to inform the outline and module development for FHS modules 6-9.   **Month 3-4**.   - FHS modules 6-9 were sent out to peer reviewers for feedback. Feedback was collated.   **Month 4**.   - Feedback was incorporated to finalise modules 6-9. Feedback was incorporated to inform the outline and module development for FHS modules 9-12. - Feedback was incorporated to finalise modules 9-12. According to feedback, a module on “Attitudes towards helping” was developed to include in pilot testing with research partners. - All FHS modules were ready for pilot testing. |
| --- |

**Supplement 2**. General training outline for EQUIP-FHS training across sites.

EQUIP-FOUNDATIONAL HELPING SKILLS

Proposed Training Schedule

Training Day 1

| ***Time*** | ***Content^*** |
| --- | --- |
| 09-10:00 | 1. Welcome 2. Ground rules 3. Introductions 4. Ice breaker and schedule |
| 10:00-10:55 | 1. Non-verbal (55 min) |
| 10:55-12:00 | 1. Break (10 min) 2. Verbal (50 min) |
| 12-13:00 | Lunch |
| 1-2:05 pm | 1. Confidentiality (65 min) |
| 14:05-15:15 | 1. Empathy, responding to feelings, & normalisation (70 min) |
| 15:15-15:25 | 1. Break (10 min) |
| 15:25-16:35 | 1. ENACT semi-assessment trainees:    1. Non-verbal & verbal communication (Item 1,2), confidentiality (Item 3), empathy (Item 6), responding to feelings & normalization (Item 5) 2. Close (10 min) |
| 16:45-17:30 | Trainers review competency results, plan remediation |

Training Day 2:

| ***Time*** | ***Content*** |
| --- | --- |
| 9-10:00 | 1. Welcome & review ground rules (10 min) 2. Remediation (50 min) |
| 10-11:40 | 1. Attitudes towards helping (90 min) 2. Break (10 min) |
| 11:45-12:40 | 1. Goal setting (55 min) 2. *Optional for trainers*:* Integrate Eliciting feedback with this module |
| 12:40-13:40 | Lunch |
| 13:40-14:50 | 1. Promoting hope (70 min) 2. *Optional for trainers**: Integrate Psychoeducation, Explanatory model with this module |
| 14:50-16:00 | 1. Assessing Suicidal Behaviours (70 min) |
| 16-16:10 | Break (10 min) |
| 16:10-17:10 | 1. ENACT semi-assessment trainees (40 min)    1. Goal setting (Item 11), Eliciting feedback (Item 15)    2. Promoting hope (Item 12)    3. Assessing suicidal behaviours (Item 7) 2. Close (10 min) |
| 17:15-18:00 | Trainers review competency results, plan remediation |

**Trainers had the option to integrate foundational helping competencies that were complementary. As such, Psychoeducation, Explanatory models, and Family Involvement were not prioritised in these trainings, but were integrated optionally by trainers.*

Training Day 3

| ***Time*** | ***Content*** |
| --- | --- |
| 9-9:10 | Welcome |
| 9:10-10:00 | Remediation or additional content from Day 1 or 2 |
| 10-11:00 | Remediation |
| 11-11:30 | Remediation |
| 11:30-13:00 | 1. Break 2. Lunch |
| 13-14:00 | End of training close  *Trainers option to give feedback* |

**Supplement 3. Figure S1.** Sequence of research and FHS training activity with ENACT competency role plays.

**
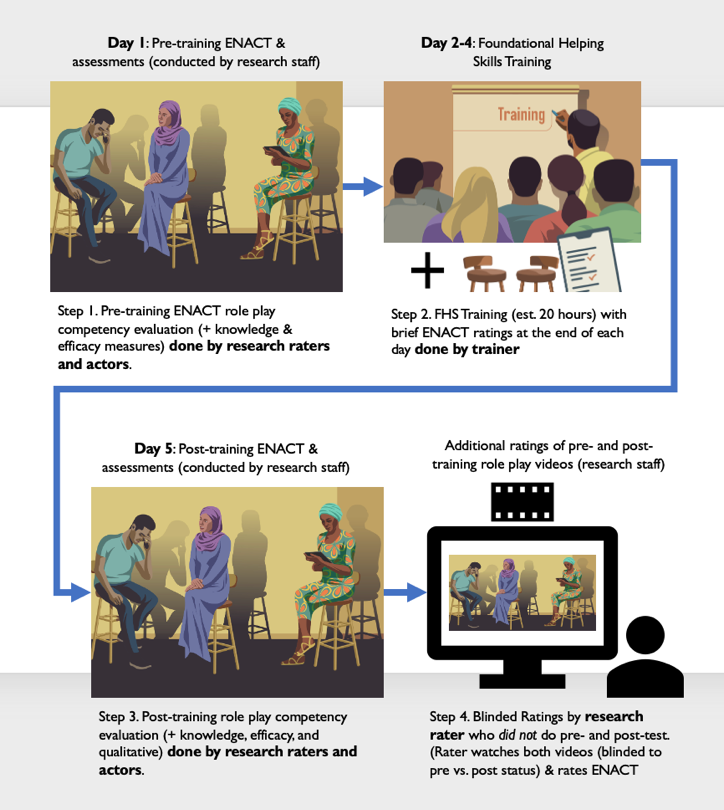
**

**Supplement 4. Table S1**. Recommended ENACT rating protocol for selecting levels based on observed behaviors.

| **LEVEL 1:**  **ANY UNHELPFUL**  **BEHAVIOUR IS SHOWN** | **LEVEL 2:**  **NO BASIC SKILLS WERE SHOWN**  **OR**  **SOME – BUT NOT ALL-**  **BASIC SKILLS SHOWN** | **LEVEL 3:**  **ALL BASIC SKILLS WERE**  **SHOWN** | **LEVEL 4:**  **ALL BASIC SKILLS WERE SHOWN**  **AND**  **ONE OR MORE ADVANCED SKILLS SHOWN** |
| --- | --- | --- | --- |
| **Mark Level 1 if:**  One or more boxes in this column are checked--even if all or some behaviors are checked in basic helping skills or advanced skills | **Mark Level 2 if:**  None or only some boxes are checked, and no harmful behaviors are seen | **Mark Level 3 if:**  All boxes are checked in the basic helping skills column, and no harmful behavior has been seen and checked off. | **Mark Level 4 if:**  All basic helping skills listed and one or more advanced skills are checked off. |

**Supplement 4.1. Inter-rater reliability processes and results (Table S2)**

Each site had between 2-6 trained raters, of which participated in an inter-rater reliability exercise prior to implementation. During implementation, for each participant, at least one rater assessed “live” (was present in-person or on Zoom), and one or more raters assessed the recorded assessment.

For each site, and for each time point (pre-implementation and live implementation) we calculated an inter-rater reliability (IRR) using an interclass correlation coefficient (ICC) with 95% confidence intervals (CI) for ENACT Level scorings (Level 1,2,3,4), and a Krippendorff’s Alpha (KAlpha) for attribute scorings. For ICC, when groups of raters did not rate the same participants, we used a one-way model to measure rater agreement at the average unit level of analysis. When groups of raters rated the same participants, we used a two-way model to measure rater agreement at the single unit level of analysis. When attribute level data was available for sites, we calculated Krippendorff’s Alpha (KAlpha) with 95% CI. For groups of raters that did not rate the same participants, we calculated separate KAlphas in groups of 2 or more raters, and calculated an average KAlpha score. Two sites (Nepal and Uganda) had missing attribute level data for pre-implementation IRR.

**Supplement 4.1 Table S2.** Inter-rater reliability (IRR) results using scores from pre-implementation and with scores from real-world implementation across sites.

|  | **IRR**  **Measure** | **Nepal** | **Peru** | **Uganda** |
| --- | --- | --- | --- | --- |
| **Pre-implementation** | Levels | *ICC*: .626 (.216, .862) | *ICC*: .69 (.477, .836) | *ICC*: .746 (.568, .890) |
|  | Attributes | Not available | *KAlpha:* .615 (.268, .824) | Not available |
| **Live Implementation** | Levels | *ICC:* .532 (.36, .669) | *ICC:* .565 (.455, .658) | ICC: .596 (.527, .661) |
|  | Attributes | *KAlpha:* .572 (.303-.724) | *KAlpha:* .533 (.380-.623) | *KAlpha:* .676 (.518, .793) |

**Supplement 4.2. Process for calculating summary scores.**

For this study, a summary rating was calculated per assessment, at the attribute and level measures, based on whether 50% or more raters marked the attribute. An algorithm supported this calculation in Excel. For example, for the attribute measures we calculated as follows for 2 or 3 raters:

Two raters, attribute measure:

=Value(IF(SUM(M48:M49)=0,"0",IF(SUM(M48:M49)=1,"1",IF(SUM(M48:M49)=2,"1")))).

Three raters, attribute measure:

=Value(IF(SUM(L2:L4)=0,”0”,IF(SUM(L2:L4)=1,”0”,IF(SUM(L2:L4)=2,”1”,IF(SUM(L2:L4)=3,”1”))).

For the level measure (Level 1, 2, 3, 4), we took a two-step process: first, an algorithm was used to calculate either the average score for 2 or more raters, for example:

Level measure:

=round(average([cell with rater1score]:[cellwithrater2,3,or4score]),0).

Second, each average summary level score was spot checked based on the summary attribute levels to ensure consistency of rating according to the ENACT rating protocol (Table S1).

**Supplement 5. Table S3.** *Implementation characteristics of the average implementation of an FHS training in Nepal (N=3), Peru (N=7), and Uganda (N=3).*

| Training implementation characteristics | Nepal | Peru | Uganda |
| --- | --- | --- | --- |
| *Total # of FHS trainings:* N | 3 | 7 | 3 |
| *Delivery format:* Hybrid, In-person, Remote | Hybrid | Remote | In-person |
| *# of trainees per training:* Mean (SD) | 14 (1.7) | 12 (1.4) | 12 (0) |
| *# of trainers per training:* N | 2 | 2 | 1 |
| *Trainee MHPSS experience (any)* | None | None | None |
| *Trainer MHPSS experience (Training, supervision, and/ or care delivery)* | 4-13 years | 3-4 years | 7+ years |
| *# of training hours per day: Mean* (SD) | 5 hours (.87) | 4.4 hours (.53) | 6 (0) |
| *Total training hours per training:* Mean (SD) | 20 hours (3.5) | 20 hours (0) | 18 (0) |
| *Total training days per training:* Mean (SD) | 4 days (0) | 4.5 days (.53) | 3 days (0) |

**Supplement 6. Table S4.** *Comparison of total scores for the ENACT harmful and helpful attributes pre- and post-training, by experience level (in-service vs. pre-service)*

| Site |  | ENACT Attributes | Mean (SD) | | Mean Difference (95% CI) | *t-*  statistic  (df) | *P-*value ^a^ |
| --- | --- | --- | --- | --- | --- | --- | --- |
|  |  |  | **Pre** | **Post** |  |  |  |
| Nepal | Pre-service (N=42) | Harmful | 4.33 (2.19) | 1 (1.86) | -3.33  (-4.03, -2.64) | -9.65 (41) | <.0001 |
|  |  | Helpful | 11.17 (5.05) | 20.43 (5.39) | 9.26  (7.76-10.77) | 12.44 (41) | <.0001 |
| Peru | Pre-service  (N=11) | Harmful | 2.45 (1.04) | .18 (.40) | -2.27 (-3.04, -1.53) | -6.83 | <.0001 |
|  |  | Helpful | 9.55 (3.11) | 17.45 (3.88) | 7.91 (4.54, 11.27) | 5.24 | <.001 |
|  | In-service  (N=70) | Harmful | 2.89 (1.23) | .74 (1.05) | -2.14 (-2.51, -1.78) | -11.75 | <.0001 |
|  |  | Helpful | 7.7 (2.82) | 13.17 (3.73) | 5.44 (4.63, 6.26) | 13.33 | <.0001 |
| Uganda | In-service (N=27) | Harmful | 2.96 (2.33) | 1.70 (1.75) | -1.26  (-2.04, -.48) | -3.32 (26) | *=.*003 |
|  |  | Helpful | 8.89 (6.08) | 12.19 (5.43) | 3.29  (1.23, 5.37) | 3.27 (26) | *=.*003 |

**Supplement 7. Figure S2.** Average number of Level 1, 2, 3, and 4 scores across participants (N=150) pre-and post-training. Error bars indicate standard deviation.


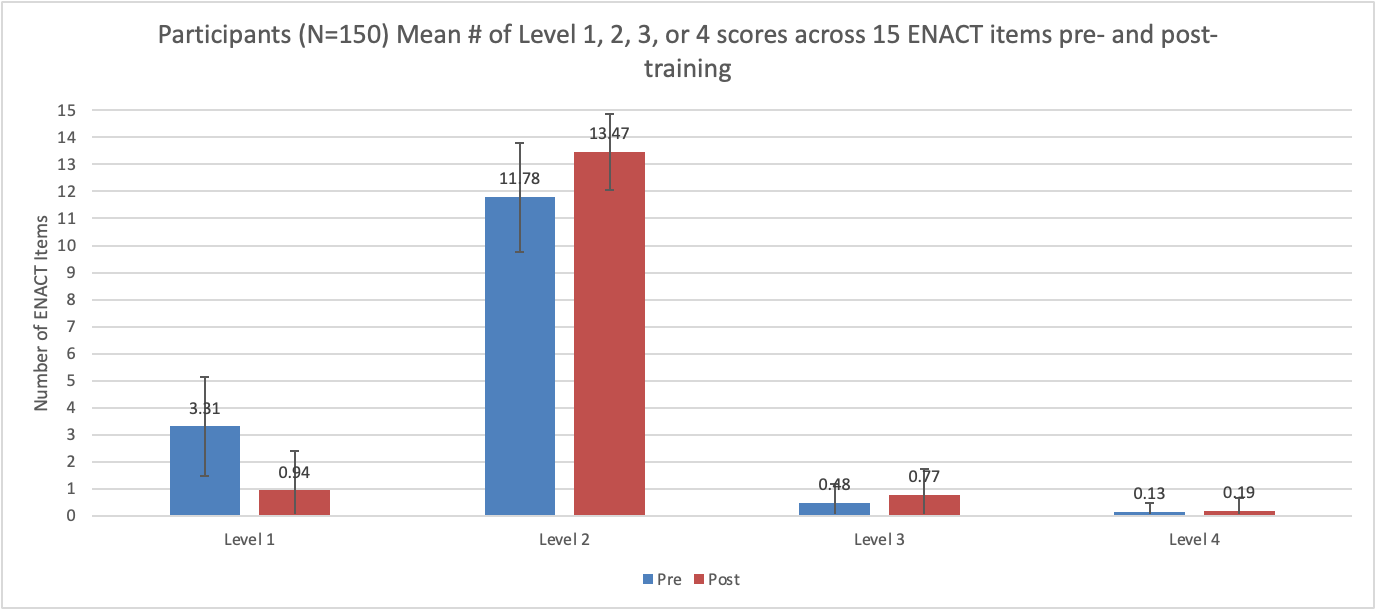


**Supplement 8. Figure S3.** Percent of participants (N=150) with Level 3,4 (competent) score per ENACT item, pre-and post-training


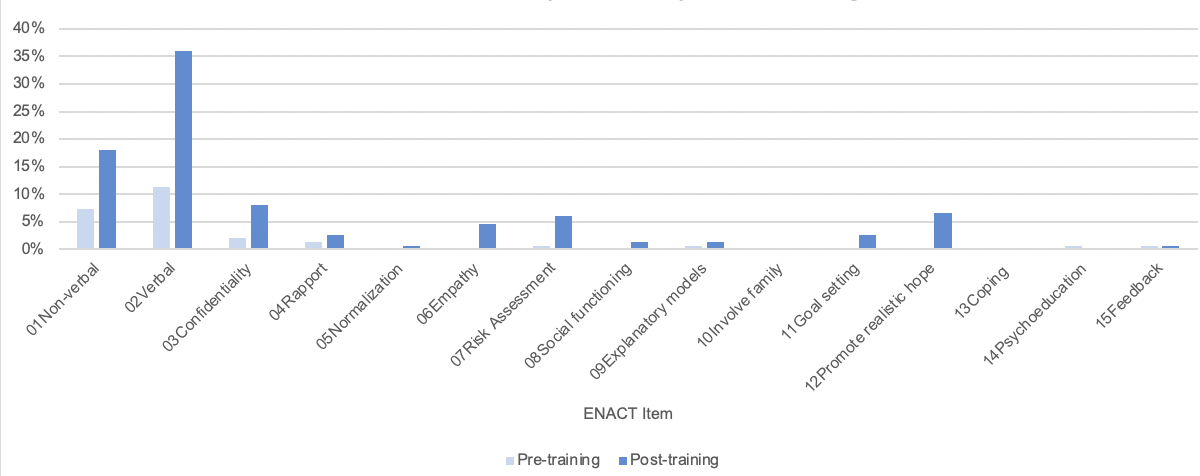


**Supplement 9. Figure S3.** Training plan by TPO Nepal that incorporates Foundational Helping Skills (FHS) training with the WHO’s Thinking Healthy Program (THP) training for providers.


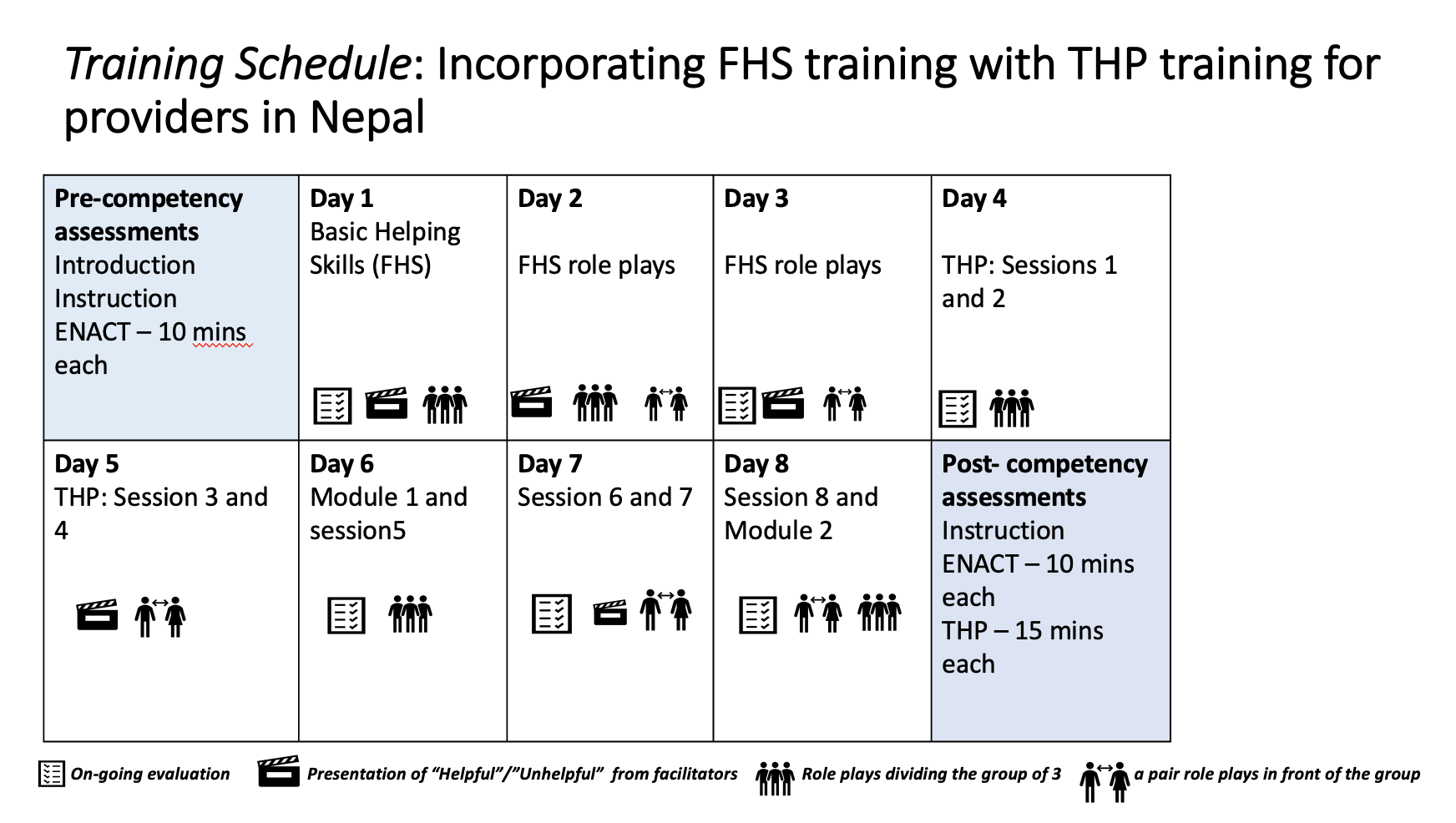

Supplement: Pedersen et al. supplementary material [file S2054425123000432sup001.docx]
